# Climate-ready crops: Unveiling the molecular dynamics of CO_2_ and glucose in plant thermotolerance

**DOI:** 10.1093/plphys/kiae131

**Published:** 2024-03-06

**Authors:** Prateek Jain

**Affiliations:** Assistant Features Editor, Plant Physiology, American Society of Plant Biologists; Department of Biology, The University of North Carolina at Chapel Hill, Chapel Hill, NC 27599-3280, USA

Globally, temperatures are surging as greenhouse gases trap atmospheric heat. According to the European Union's Copernicus Climate Change Service, 2023 was an average 1.48°C warmer than the pre-industrial temperature of 1850 to 1900 (https://climate.copernicus.eu). Extremely high temperatures and excessive greenhouse gases adversely affect both human and plant health. This situation is causing global concern as it interferes with crop productivity and poses a challenge to food security. It becomes necessary to find solutions to address high temperatures and greenhouse gases such as CO_2_ while boosting crop yield and productivity. The genetic engineering and synthetic biology approaches could be helpful in “reprogramming” biological systems to help plants sustain adverse environmental conditions, such as high heat and excess greenhouse gases.

In this issue of *Plant Physiology*, Wang et al. (2024) have reported that excessive CO_2_ and external glucose (Gluc) treatment can contribute to thermotolerance in tomato plants (Wang et al. 2024). Elevated CO_2_ generally causes higher levels of apoplastic Gluc, which is an energy source and signaling molecule (Häusler et al. 2014; Thompson et al. 2017). How can CO_2_ and Gluc improve the thermotolerance? To answer this question, the authors first hypothesized that CO_2_ might provide thermotolerance in tomato plants and showed that high levels of CO_2_ (800 *μ*mol mol^−1^) make plants more heat resistant compared to ambient CO_2_ (400 *μ*mol mol^−1^) when grown under high temperature (45°C) (Fig. A). One might wonder how excessive CO_2_ helps plants become heat resistant and how it coordinates with Gluc in downstream signaling. To establish a link between elevated CO_2_-modulated thermotolerance and Gluc, the relative Gluc levels in the apoplast and whole cells were analyzed under elevated CO_2_ conditions. The authors showed that elevated CO_2_ treatment caused a 1.93-fold increase in the apoplastic Gluc levels. However, the question remains as to whether Gluc treatment can provide thermotolerance in tomato plants. Wang et al. (2024) found that tomato plants sprayed with exogenous Gluc, even at ambient CO_2_, have higher heat resistance and a stable photochemical quantum yield of PSII (Fv/Fm) compared to wild type (Wang et al. 2024). This highlights the crucial roles of CO_2_ and Gluc in thermotolerance.

**Figure.**
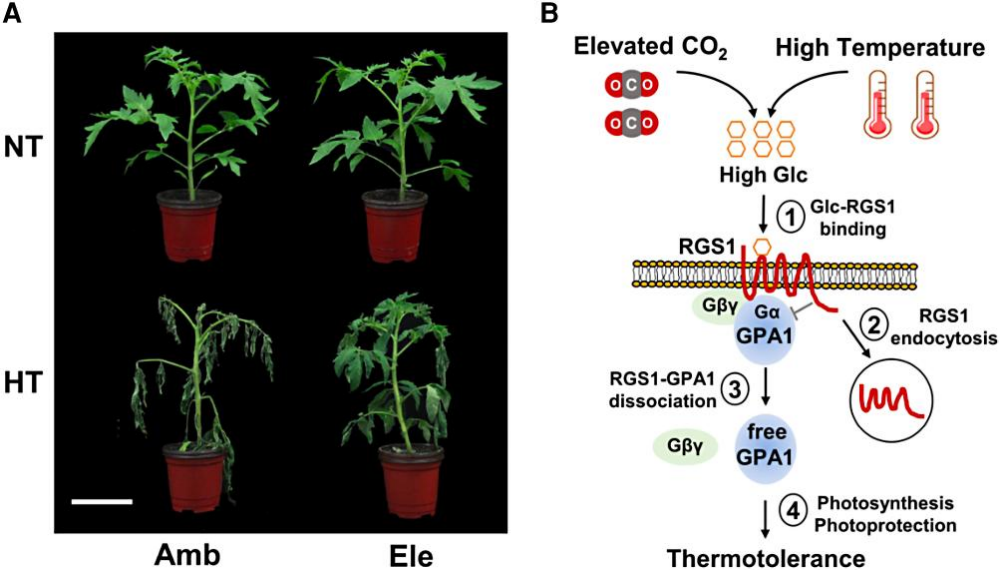
**A)** Tomato plants showing thermotolerance at elevated level of CO_2_. **B)** A working model showing Gluc-G protein signaling pathway regulates plant resilience under high temperature and elevated CO_2_ in tomato. Amb, ambient CO_2_ (400 *μ*mol mol^−1^); Ele, elevate CO_2_ (800 *μ*mol mol^−1^); HT, higher temperature (45°C); NT, normal temperature (25°C) (Wang et al. 2024).

Next, the authors deciphered the underlying mechanism in Gluc-mediated thermotolerance. In *Arabidopsis*, the regulator of G protein signaling 1 (RGS1)/heterotrimeric G protein pathway has been reported as a Gluc sensor (Uranao et al. 2012; Wang et al. 2022). Wang et al. (2024) demonstrated a direct interaction between RGS1 and Gluc using a cell-based biolayer interferometry (cBLI) assay and found that RGS1 acts as a negative regulator during heat stress in tomatoes (Wang et al. 2024). Previously, Fu et al. (2014) showed that RGS1 binds to a Gα protein (AtGPA1), a subunit of G-protein, and Gluc causes the dissociation of the RGS1-GPA1 complex in *Arabidopsis* (Urano et al. 2012; Fu et al. 2014). Therefore, the authors performed a bimolecular fluorescence complementation (BiFC) assay and observe the interaction between RGS1 and GPA1. In addition, using luciferase complementation (Split Luc) assay, the authors confirmed that Gluc treatment causes the dissociation of the RGS1-GPA1 dimeric complex in tomatoes. Further, the authors have generated the gene editing lines of *gpa1* and showed that *gpa1* tomato lines have reduced thermotolerance. It confirms the direct role of *GPA1* in thermotolerance.

Overall, the work of Wang et al. 2024 is notable, as it establishes Gluc treatment and elevated CO_2_ as tools to provide thermotolerance in plants. The authors pursued an intriguing line of research, showing that Gluc and elevated CO_2_ directly affect the G-protein signaling cascade to promote heat resistance in crop plants. The authors found similarities in the molecular mechanisms of RGS1 and GPA1 under heat stress in *Arabidopsis* and tomatoes (Fig. B). Expanding this analysis to key crops that produce fruits and cereals could enrich our ability to develop heat tolerance and support higher yield and productivity.

## Dive Curated Terms

The following phenotypic, genotypic, and functional terms are of significance to the work described in this paper:

RGS1 Gramene: AT3G26090
RGS1 Araport: AT3G26090
